# Lipophilic aroylhydrazone chelator HNTMB and its multiple effects on ovarian cancer cells

**DOI:** 10.1186/1471-2407-10-72

**Published:** 2010-02-25

**Authors:** Kyu Kwang Kim, Thilo S Lange, Rakesh K Singh, Laurent Brard

**Affiliations:** 1 Molecular Therapeutics Laboratory, Program in Women's Oncology, Department of Obstetrics and Gynecology, Women and Infants' Hospital of RI, Alpert Medical School of Brown University, Providence, RI 02905, USA; 2 Division of Biology and Medicine, Brown University, Providence, RI 02912, USA

## Abstract

**Background:**

Metal chelators have gained much attention as potential anti-cancer agents. However, the effects of chelators are often linked solely to their capacity to bind iron while the potential complexation of other trace metals has not been fully investigated. In present study, we evaluated the effects of various lipophilic aroylhydrazone chelators (AHC), including novel compound HNTMB, on various ovarian cancer cell lines (SKOV-3, OVCAR-3, NUTU-19).

**Methods:**

Cell viability was analyzed via MTS cytotoxicity assays and NCI60 cancer cell growth screens. Apoptotic events were monitored via Western Blot analysis, fluorescence microscopy and TUNEL assay. FACS analysis was carried out to study Cell Cycle regulation and detection of intracellular Reactive Oxygen Species (ROS)

**Results:**

HNTMB displayed high cytotoxicity (IC50 200-400 nM) compared to previously developed AHC (oVtBBH, HNtBBH, StBBH/206, HNTh2H/315, HNI/311; IC50 0.8-6 μM) or cancer drug Deferoxamine, a hexadentate iron-chelator (IC50 12-25 μM). In a NCI60 cancer cell line screen HNTMB exhibited growth inhibitory effects with remarkable differences in specificity depending on the cell line studied (GI50 10 nM-2.4 μM). In SKOV-3 ovarian cancer cells HNTMB treatment led to chromatin fragmentation and activation of the extrinsic and intrinsic pathways of apoptosis with specific down-regulation of Bcl-2. HNTMB caused delayed cell cycle progression of SKOV-3 through G2/M phase arrest. HNTMB can chelate iron and copper of different oxidation states. Complexation with copper lead to high cytotoxicity via generation of reactive oxygen species (ROS) while treatment with iron complexes of the drug caused neither cytotoxicity nor increased ROS levels.

**Conclusions:**

The present report suggests that both, non-complexed HNTMB as a chelator of intracellular trace-metals as well as a cytotoxic HNTMB/copper complex may be developed as potential therapeutic drugs in the treatment of ovarian and other solid tumors.

## Background

The current treatment of a variety of tumors, including ovarian cancer, relies on organometallic platinum compounds. Ovarian cancer is the leading cause of death from gynecologic malignancies and ranks second among newly diagnosed gynecological cancers in the United States [1,2]. Although most women will initially respond to cytoreductive surgery and adjuvant paclitaxel-based and platinum-based chemotherapy, the majority will experience disease recurrence. While re-treatment with a platinum-based agent is possible for some women, overall response rates to second line chemotherapeutic agents are 15-30% and treatment of recurrent ovarian carcinoma is mainly directed at palliation [3-6]. Treatment strategies against tumors that developed resistance to standard chemotherapeutic agents, most notably platinum analogs, include non-platinum drugs with increased activity and response rates. Chelating drugs and chelator metal complexes are used for the prevention, diagnosis and treatment of cancer and chelating compounds with high affinity for iron or copper have been suggested as potential anti-tumor agents [7]. In previous studies the effects of chelating drugs were often linked solely to their capacity to complex iron while the potential complexation of other trace metals was not discussed or analyzed. One rationale for the anti-tumor activity of chelators is a higher Fe utilization by cancer cells and often elevated concentrations of trace metals, particularly of copper, in tumor patients [8-10].

Copper chelators such as D-penicillamine, trientine, tetrathiomolybdate are currently being used in the treatment of copper-overload disorders such as Wilson's disease. Copper complexes such as 8-hydroxyquinoline derivatives, pyrrolidine dithiocarbamate and clioquinol have been reported to be cytotoxic against cancer cells [11,12]. Copper is an essential cofactor for several extracellular and a multitude of intracellular enzymes and plays a pivotal role in cellular metabolism including energy production (cytochrome c oxidase), intracellular metal detoxification (Cu(I)-glutathione-complex mediated metallothionein activity), iron detoxification (via ceruloplasmin), connective tissue formation (lysyl oxidase), and antioxidant defense system [Cu/Zn superoxide dismutase (SOD)] against ROS [13,14]. ROS are tightly regulated in balance with cellular defensive antioxidants, such as catalase and SOD, and can participate in a multitude of cellular functions (e.g., signal transduction, platelet aggregation, immune system control, production of energy, phagocytosis, regulation of cellular growth, metabolism of xenobiotics) [15]. When generated excessively or when antioxidant function is disturbed, ROS can be cytotoxic through the oxidation of biomolecules (e.g., membranes, enzymes, carbohydrates, DNA). ROS have been implicated in cancer initiation, promotion and progression [16,17]. Cancer cells, presumably through mitochondria dysfunction and increased metabolism, generate a relatively high level of ROS and modulation of cellular ROS has been suggested as a strategy to selectively target cancer cells over normal cells [18,19].

Iron chelators suggested as potential anti-tumor agents include deferoxamine (DFO) [20], deferiprone and deferasirox [21], tachpyridine [22], triapine [23] and O-trensox [24]. Iron is an essential component of many biological molecules including hemoglobin, myoglobin, ribonucleotide reductase (RR), cyclooxygenases, lipoxygenases, iron-sulfur proteins and hydroxylating enzymes [22,25]. An elevated level of iron has been linked to tumor risk [26] and the growth of neoplastic cells due to iron's catalytic effect on the formation of hydroxyl radicals and the suppression of host defense cell activity [27]. Neoplastic cells display an elevated expression of transferrin and its receptor as well as a high rate of iron internalization thereby justifying the development of chelating compounds for cancer therapy [28,29]. Accordingly, cancer cell death can be induced by depleting the intracellular iron pool as shown by H-ferritin expression in ovarian cancer cells [30]. Targeting tumors with chelating agents in an attempt to alter cellular iron homeostasis or metabolism is a promising treatment approach [31].

The objective of the present study was to investigate the cytotoxic potential of 6- lipophilic aroylhydrazone chelators (*AHCs*) in ovarian cancer cell lines. The present report suggests that newly designed compound *HNTMB *displays cytotoxic properties superior to other *AHCs *and to iron-chelator deferoxamine (included as control [20]). The mode of action in SKOV-3 ovarian cancer cells relies on the generation of ROS, caspase activation, Bcl-2 reduction, DNA degradation and G2/M phase cell cycle block. *HNTMB *when complexed with copper (I) or (II) but not with Fe(II) or (III) generates ROS and is highly cytotoxic. In summary, *HNTMB *may serve as a potential therapeutic drug and an alternative to platinum drugs in the treatment of ovarian cancer.

## Methods

### Synthesis and Materials

Deferoxamine mesylate (DFO) was purchased from Sigma-Aldrich (St. Louis, MO). *AHC *compounds oVtBBH [32], HNtBBH [33], StBBH/206, HNTh2H/315 and HNI/311 [34,35] have been described previously. All *AHCs *were synthesized as follows using the Schiff base condensation method. Briefly, each aldehyde was combined with an equimolar amount of the corresponding hydrazide in ethanol, stirred and refluxed. The resultant materials were collected by filtration to afford the desired product and were further purified by re-crystallization (HNI, StBBH and oVtBBH). The compounds were characterized by ^1^H nuclear magnetic resonance spectroscopy (^1^H NMR) and mass spectrometry. Salicylaldehyde-4-t-butylbenzoyl hydrazone (StBBH/206): yield 92%. Analytical data; ^1^H NMR [dimethyl sulfoxide (DMSO)-d_6_, δ] 12.0 (1H), 11.3 (1H), 8.6 (1H), 7.9 (2H), 7.5 (3H), 7.3 (1H), 6.9 (2H), 1.3 (9H). FAB-MS m/z (nitrobenzyl alcohol/NaI matrix) calculated for C_18_H_20_N_2_O_2_, 296.15 [M^+^], found 319 [M+Na]^+^. 2-hydroxy-1-naphthylaldehyde-4-t-butylbenzoyl hydrazone (HNtBBH): yield 71%. Analytical data; ^1^H NMR (DMSO-d_6_, δ) 12.8 (1H), 12.1 (1H), 9.5 (1H), 8.2 (1H), 7.9 (4H), 7.6 (3H), 7.4 (1H), 7.2 (1H), 1.3 (9H). FAB-MS m/z (nitrobenzyl alcohol/NaI matrix) calculated for C_22_H_22_N_2_O_2_, 346.2 [M^+^], found 369.2 [M+Na]^+^. 3-Methoxysalicylaldehyde-4-t-butylbenzoyl hydrazone (oVtBBH): yield 69%. Analytical data; ^1^H NMR (DMSO-d_6_, δ;) 12.0 (1H), 11.09 (1H), 8.6 (1H), 7.9 (2H), 7.5 (2H), 7.1 (1H), 7.0 (1H), 6.8 (1H), 3.8 (3H) 1.3 (9H). FAB-MS m/z (nitrobenzyl alcohol/NaI matrix) calculated for C_19_H_22_N_2_O_3_, 326.2 [M^+^], found 349.1 [M+Na]^+^. 2-hydroxy-1-naphthylaldehyde 3,4,5-trimethoxy-benzoylhydrazone (*HNTMB*): yield 67%. ^1^H NMR (DMSO-d_6_, δ) 12.8 (1H), 12.1 (1H), 9.5 (1H), 8.3 (1H), 7.9 (2H), 7.6 (1H), 7.3 (4H), 3.9 (6H), 3.8 (3H). FAB-MS m/z (nitrobenzyl alcohol/NaI matrix) calculated for C_21_H_20_N_2_O_5_, 380.14 [M^+^], found 403.3 [M+Na]^+^. 2-hydroxy-1-naphthylaldehyde isonicotinoyl hydrazone (HNI/311): 58% yielded. Analytical data; ^1^H NMR (DMSO-d_6_, δ) 12.5 (1H), 12.4 (1H), 9.5 (1H), 8.8 (2H), 8.3 (1H) 7.9 (4H) 7.6 (1H) 7.4 (1H) 7.2 (1H). ES-MS calcd for C_17_H_13_N_3_O_2_, 291.1 [M^+^], found 292.3 [M+1]^+^. 2-Hydroxy-1-naphthylaldehyde 2-thiophenecarboxy hydrazone (HNTh2H/315): yield 59.2%. Analytical data; ^1^H NMR (DMSO-d_6_, δ) 12.6 (1H), 12.2 (1H), 9.4 (1H), 8.3 (1H), 7.9 (4H), 7.6 (1H), 7.4 (1H), 7.2 (2H). FAB-MS m/z (nitrobenzyl alcohol/NaI matrix) calculated for C_16_H_12_N_2_O_2_S, 296.06 [M^+^], found 319.2 [M+Na]^+^.

### Cell culture and cell viability assay

SKOV-3 and OVCAR-3 (human ovarian adenocarcinomas) were purchased from ATCC (Manassas, VA). Cells were grown in Dulbecco's Modified Eagle's Medium (DMEM) supplemented with bovine calf serum or fetal calf serum (10% for SKOV-3, 20% for OVCAR-3). NUTU-19 rat epithelial ovarian cancer cells derived from Fischer 344 rats [36], a gift from Dr. G. Scott Rose (Section of Gynecologic Oncology, Cleveland Clinic Foundation, Cleveland, OH 44195, USA), were cultured in RPMI 1640 supplemented with 10% fetal bovine serum. Cells were cultured in the presence of 100 unit/ml penicillin G and streptomycin at 37°C with 5% of CO_2 _in a humidified incubator. Viability of cell lines before and after drug treatment was determined by the CellTiter 96^® ^AQueous One Solution Assay (Promega Corp, Madison, WI) following the manufacturer's recommendations with suitable modifications. This colorimetric assay is based on the ability of mitochondria to reduce a substrate [MTS, 3-(4,5-dimethylthiazol-2-yl)-5-(3-carboxymethoxyphenyl)-2-(4-sulfophenyl)-2H-tetrazolium] into a soluble formazan product quantified by measuring the absorbance at 490 nm. The resulting OD is directly proportional to the number of living cells [37]. Briefly, cells were seeded into a 96-well microtiter plate at 5,000 cells/100 μl per well in complete medium, allowed to attach at 37°C with 5% of CO_2_, in a humidified incubator and cells were treated with complete medium containing drugs originally dissolved in dimethyl sulfoxide (DMSO). Concentrations of the drugs are indicated for the respective experiment; the final concentration of the vehicle did not exceed more than 0.2% (v/v). To determine the cytotoxicity when complexed with Fe(II), Fe(III), Cu(I) or Cu(II), *HNTMB *was combined with the respective metal salt (FeCl_2_, FeCl_3_, CuCl or CuCl_2_) (5 mM stock solutions in DMSO). Complexes were formed by combining stock solutions of salts and chelator to 1000× the desired final assay concentration in DMSO, incubated at 37°C for 30 min before further dilution in complete medium for cell treatment. During the last 4 h of incubation at 37°C in a cell culture incubator, the MTS reagent was added at a 1:10 dilution to the medium. After the treatment period (as indicated) absorbance was measured at 490 nm in an ELISA plate reader (Thermo Labsystems, Waltham, MA). Experiments were performed in triplicates; data are expressed as the mean of the triplicate determinations (X ± SD) of a representative experiment in % of absorbance by samples with untreated cells [=100%].

### NCI 60 cancer cell line assay

*HNTMB *was screened through the National Cancer Institute (NCI) Developmental Therapeutics Program (DTP) 60 human cancer cell line panel under the *In Vitro *Cell Line screening Project (IVCLSP). Briefly, cells (5,000 to 40,000 cells/well depending on the cell line studied) were inoculated into 96 well microtiter plates in 100 μl complete RPMI1640 medium (5% FBS and 2 mM L-glutamine) and incubated 24 h prior to addition of *HNTMB*. *HNTMB *was added and the plates were incubated at 37°C, 5% CO_2_, 95% air, and 100% relative humidity for an additional 48 h. Upon the addition of 50 μl of cold TCA (10% TCA) the assay was terminated and incubated for 60 min at 4°C to fix the cells. The supernatant was discarded, and the plates washed five times with water and air dried. Sulforhodamine B solution (100 μl) at 0.4% (v/v) in 1% acetic acid was added to each well, and plates incubated for 10 min at room temperature. After staining, unbound dye was removed by washing five times with 1% acetic acid and the plates were air dried. Bound stain was subsequently solubilized with 10 mM trizma base, and the absorbance was read on an automated plate reader at a wavelength of 515 nm. Using absorbance measurements [time zero (Tz), control growth (C), and test growth in the presence of drug at the drug concentration (Ti)], the percentage growth was calculated. Percentage growth inhibition was calculated as: [(Ti-Tz)/(C-Tz)] × 100 for concentrations for which Ti >/= Tz, [(Ti-Tz)/Tz] × 100 for concentrations for which Ti < Tz.

### Morphological Studies

To assess morphological changes and chromatin condensation of SKOV-3 cells undergoing apoptosis following treatment with an iron-chelator, cells were stained with 4',6-diamidino-2-phenylindole (DAPI) and examined by fluorescence microscopy. Cells were seeded into compartments of 8-well Lab-Tek^® ^II chamber slide (Nalge Nunc International, Naperville, IL), allowed to attach onto the slide and deprived of serum for 18 h prior to the incubation with 0.2 μM *HNTMB *for 24 h. The cells were then washed with 1×PBS with Calcium/Magnesium (Invitrogen, Carlsbad, CA), fixed and permeablized by 2% paraformaldehyde solution with 0.2% Trition-X-100 in 1×PBS for 30 min at room temperature. The fixed cells were washed with 1×PBS. After removal of the upper chamber and silicone lining the cells were stained and mounted at room temperature with Vectashield^® ^Mounting Medium for Fluorescence with DAPI (Vector Laboratories). Once the excess amount of mounting medium was removed, the slide was examined under the fluorescence microscope (ECLIPSE TE 2000-E, Nikon Inc.).

### TUNEL Assay

DNA fragmentation was detected using the DeadEndTM Fluorometric TUNEL System assay (Promega, Madison, WI) according to the manufacturer's recommendations. Cells (4 × 10^5^/well) were seeded into 8-well Lab-Tek^® ^11 chamber slide (Nalge Nunc International, Naperville, IL), treated with 0.2 μM *HNTMB *and the assay carried out as described previously [38]. Fluorescence of apoptotic cells (green; labeling of DNA nicks by fluorescein-12-dUTP) and of chromatin (red; staining of chromatin with propidium iodide) was detected by fluorescence microscopy with an inverted microscope (Nikon Eclipse TE2000-E) and a 20× objective. Four randomly chosen microscopic fields were captured.

### Western Blot Analysis

SKOV-3 cells (1.5 × 10^6 ^per dish) were seeded into 100 mm cell culture dishes allowed to attach overnight and serum-deprived for 18 h prior to the treatment under the condition as mentioned. Following treatment, cells were processed as described previously [39] in Cell Extraction Buffer (BioSource International, Inc., CA.) supplemented with protease inhibitor cocktail and phenylmethylsulfonyl fluoride (Sigma-Aldrich) according to the manufacturers' recommendations. Lysates were incubated at 4°C for 30 min, sonicated (10 pulses 5 sec), centrifuged at 14,0000 g for 10 min, and the protein concentration of the supernatant quantified (BioRad protein estimation kit, Hercules, CA). Protein electrophoresis was performed by using the NuPAGE^® ^Gel system (Invitrogen, Carlsbad, CA). Briefly, each lysate sample was mixed with LDS sample buffer and sample reducing buffer, incubated at 70°C for 10 min, loaded (50 μg/sample) and separated by using Xcell SureLockTM mini-cell electrophoresis system (Invitrogen, Carlsbad, CA) on NuPAGE^® ^4-12% Tris-Bis Gel in NuPAGE^® ^MES SDS running buffer, transferred onto a PVDF membrane, blocked with 5% nonfat dry milk in PBS-Tween and probed against various primary antibodies (dilution 1:1000, all from rabbit: PARP #9541, XIAP #2045, caspase-3 #9661, caspase-7 #9491, caspase-9 #9501 (Asp330/37 kD fragment) or #9505 (Asp315/35 kD fragment), caspase-8 #9496, Bcl-xL #2762, Bid #2002; Cell Signaling Technologies, Danvers, MA), Bcl-2 #551107 (BD Biosciences, San Jose, CA) or mouse beta-actin #sc-47778 or GAPDH #sc-47724 antibody (Santacruz Biotechnology, Santa Cruz, CA; dilution 1:2000). The bands were visualized using horseradish peroxidase-conjugated secondary antibody (Amersham-Pharmacia Biotech, Piscataway, NJ), followed by enhanced chemiluminescence (Upstate, Waltham, MA) and documented by autoradiography (F-Bx810 Film, Phenix, Hayward, CA).

### Cell cycle analysis

Cell cycle analysis and quantification of apoptosis was carried out by flow cytometry. SKOV-3 (1.0 × 10^6^) cells were seeded into 100 mm cell culture dishes, allowed to attach overnight and treated for 48 h. At the end of the incubation period, cells were scraped off and transferred into 15 mL polypropylene centrifuge tubes along with the medium. Culture dishes were then washed once with 1× PBS, combined in the same tube. After centrifugation (250 g, 5 min) cells were fixed by adding the ice-cold 70% ethanol solution gradually. The cells were stained in the buffer containing propidium iodide (100 μg/ml), sodium citrate (1 mg/ml) and Triton-X-100 (3 μL/ml) for 30 min at 37°C in the dark. Data was acquired on a BD FACSort flow cytometer using CellQuest software (BD Immunocytometry Systems, San Jose, CA) and analyzed by using ModFit LT software (Verity Software House, Inc., Topsham, ME). Ten thousand events were analyzed for each sample. Appropriate gating was used to select the single cell population SKOV-3 cells. The same gate was used on all samples, ensuring that measurements were conducted on a standardized cell population.

### Detection of intracellular Reactive Oxygen Species (ROS)

Detection of intracellular ROS after SKOV-3 treatment with non-complexed or complexed *HNTMB *(see Viability assay section) was measured by flow cytometry using carboxy-H2DCFDA dye (Invitrogen, Carlsbad, CA) as a probe. Carboxy-H2DCFDA is the acetylated form of a reduced fluorescein derivative that is cell-permeable and non-fluorescent. Once the acetate groups are cleaved by intracellular esterase activities, this compound becomes charged and better retained within the cell as compared to its lipophilic parent compound. In the presence of a cellular oxidant, the compound is oxidized and produces green-fluorescence that is detected by flow cytometry. This dye detects the following ROS: hydrogen peroxide (H_2_O_2_), hydroxyl radical (HO^•^), and peroxyl radical (ROO^•^). SKOV-3 (1.0 × 10^6^) cells were seeded into 100 mm cell culture dishes, allowed to attach overnight and treated under the condition as indicated. Following treatment, cells were further incubated with 25 μM of carboxy-H2DCFDA for 30 min at 37°C with 5% CO_2 _in a humidified incubator. Cells were harvested by trypsinization, centrifuged, then washed once with PBS and suspended in PBS. Data was acquired on a BD FACSort flow cytometer using CellQuest software (BD Immunocytometry Systems, San Jose, CA) and analyzed by using ModFit LT software (Verity Software House, Inc., Topsham, ME).

### Data Analysis

Mean and standard deviation (SD) were calculated. Mean differences were determined by Student's t-test or determined by one-way ANOVA, using the Newman-Keuls test to account for multiple comparisons in post hoc analyses, except were indicated. Software used for these analyses was STATA 9.0 (StataCorp, College Station, TX.

## Results

### Cytotoxic effect of aroylhydrazone chelators on ovarian cancer cell lines

**Figure 1:**
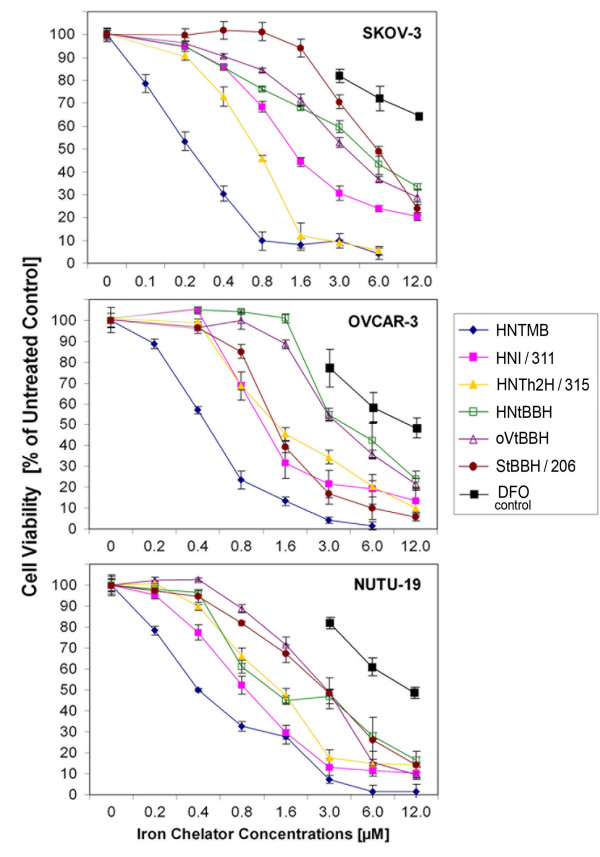
**Cytotoxic effect of aroylhydrazone chelators (*AHC*) in comparison to DFO in ovarian cancer cells**. The cytotoxicity of chelators on human ovarian cancer cells (SKOV-3, OVCAR-3) and a rat ovarian cancer cells (NUTU-19) was evaluated by using the MTS viability assay as described (Materials and Methods). The cells were treated for 72 h with varying concentrations of various *AHCs *or DFO as control. *HNTMB *displayed the highest cytotoxicity (IC_50 _= 0.2-0.8 μM depending on the cell line studied). Experiments were performed in triplicates; data are expressed as the mean of the triplicate determinations (X ± SD) of a representative experiment in % cell viability of untreated cells [100%].

**Figure 2:**
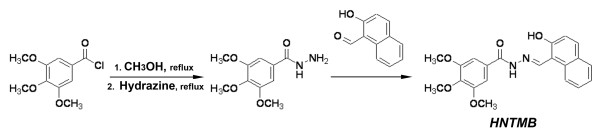
**Structure of *HNTMB***. Synthetic scheme (Schiff base condensation method; for synthesis details see Materials and Methods) and structure of *HNTMB*.

In an initial approach to analyze the potential of a variety of lipophilic iron-chelators of the *AHC *class as anti-cancer drugs we performed viability assays employing two human platinum-resistant ovarian cancer cell lines (SKOV-3 and OVCAR-3) a rat ovarian cancer cell line (NUTU-19). The cytotoxic activity of each compound was measured by a colorimetric MTS assay (see Material and Methods), based on the reduction of a tetrazolium compound in active cell metabolism. DFO was included as a control compound. After cell treatment for 72 h all six *AHC *tested showed more potent cytotoxic effects (Fig. 1) than DFO (IC_50 _of 25 μM for SKOV-3, 12 μM for OVCAR-3 and NUTU-19) in all tested ovarian cancer cell lines. Novel compound *HNTMB *(Fig. 2), displayed superior cytotoxicity (72 h treatment, IC_50 _of 200 nM for SKOV-3, 400 nM for OVCAR-3 and NUTU-19) (Fig. 1) when compared to all other 5 *AHC *tested (oVtBBH, HNtBBH, StBBH/206, HNTh2H/315, HNI/311) which were highly cytotoxic only at higher drug concentrations with IC_50 _values between 800 nM and 6 μM depending on the cell line or compound studied.

### HNTMB displays differential effects on the growth of various human cancer cell lines

**Figure 3:**
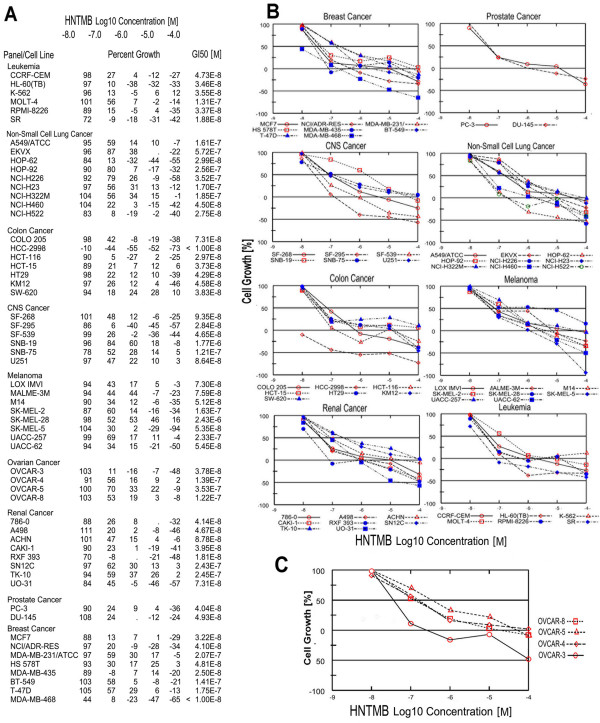
**Cell growth after *HNTMB *treatment in NCI60 cancer cell line screen**. Cells were treated in 96 well plates with *HNTMB *or vehicle and cell growth of the TCA fixed treated and untreated cells assessed (Material and Methods) after 48 h with Sulphorhodamine-B (SRB) solution and absorbance read at 515 nm.

The NCI-DTP performed a screen on *HNTMB *as a growth suppressor against a panel of 60 human cancer cell lines. The *HNTMB *concentrations representing 50% growth inhibition (GI_50_, Fig. 3A) were determined by using the dose-response growth curves of OVCAR ovarian cancer cell lines and other cancer cells (colon, lung, melanoma, leukemia, renal, prostate, central nervous system) against *HNTMB *concentrations ranging from 10 nM to 100 μM) (Fig. 3A, B). *HNTMB *exhibited growth inhibitory effects against all cell lines tested with remarkable differences in specificity in a 240 fold range of GI_50 _values (between 10 nM and 2.4 μM depending on the cell line studied). HCC-2998 (colon) and MDA-MB-468 (breast) cancer cell lines appeared to be the most sensitive toward the *HNTMB *treatments (GI_50 _≤ 10 nM). Relatively strong growth inhibitory effects (with GI_50 _values less than 50 nM) of *HNTMB *were observed in 5 out of 6 leukemia cells (CCRF-CEM, HL-60(TB), K-562, RPMI-8226 and SR), 3 out of 9 non-small cell lung cancer cells (HOP-62, NCI-H460 and NCI-H522), 6 out of 7 colon cancer cells (HCC-2998, HCT-116, HCT-15, HT29, KM12 and SW-620), 2 out of 6 CNS cancer cells (SF-295 and SF-539), 4 out of 8 renal cancer cells (786-0, A498, CAKI-1 and RXF 393), 2 out of 2 prostate cancer (PC-3 and DU-145) and 5 out of 8 breast cancer cells (MCF-7, NCI/ADR-RES, HS 578T, MDA-MB-435 and MDA-MB-468). Among ovarian cancer OVCAR-3 (GI_50 _= 38 nM) displayed the highest sensitivity when compared to OVCAR-4, -5, and -8 (Fig. 3C).

### Morphological changes, DNA fragmentation in SKOV-3 ovarian cancer cells after HNTMB treatment

**Figure 4:**
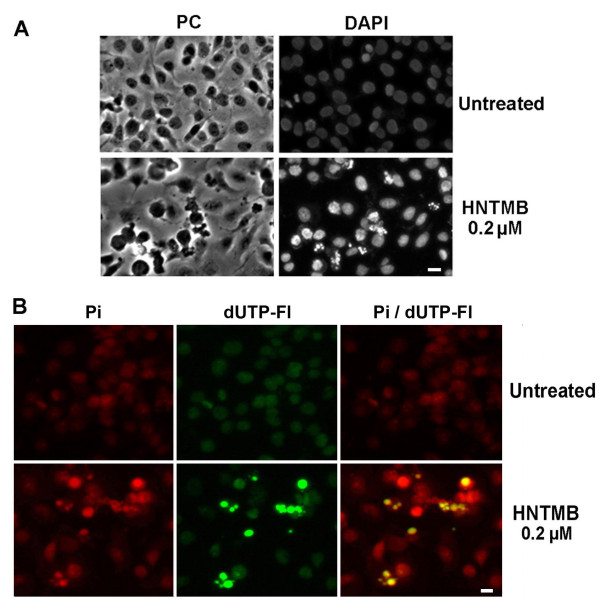
**Morphology changes and DNA fragmentation in ovarian cancer cells after *HNTMB *treatment**. **(A) Morphological changes following *HNTMB *treatment**. SKOV-3 cells were treated for 24 h with 0.2 μM *HNTMB*, fixed and stained with 4'-6-Diamidino-2-Phenylindole (DAPI) as described (Materials and Methods) before mounting. Microscopy was carried out (Nikon Eclipse TE2000-E inverted microscope, 20× objective) and representative images were taken. Bar = 10 μm. **(B) TUNEL assay**. SKOV-3 cells were treated with 0.2 μM of *HNTMB *for 24 h. Labeling of DNA nicks with fluorescein-12-dUTP and chromatin counterstaining with propidium iodide was carried out as described (Materials and Methods). Representative images were taken, apoptotic stain (FL-dUTP, green) and nuclear stain (Pi, red) overlaid; TUNEL positive nuclei due to DNA fragmentation appear as yellow areas. Bar = 10 μm.

Cells undergoing apoptosis display characteristic changes in their morphology including plasma membrane blebbing, cell shrinkage, condensation and fragmentation of the nucleus and the formation of apoptotic bodies. In order to distinguish whether cell death in ovarian cancer cells upon *HNTMB *treatment is due to apoptosis, we first examined the morphologic changes of SKOV-3 cells after drug treatment. Nuclear staining using 4',6-diamidino-2-phenylindole (DAPI), a fluorescent DNA-binding dye, revealed that ~40% of SKOV-3 cells treated with *HNTMB *exhibited apparent nucleus condensation and fragmentation upon *HNTMB *treatment (0.2 μM, 24 h) whereas the untreated control cells were not affected (Fig. 4A).

Another hallmark feature of cells undergoing apoptosis is often characterized by chromatin fragmentation e.g. cleavage of genomic DNA to 180-200 bp fragments. This event within individual cells can be analyzed by terminal deoxynucleotidyl transferase-mediated dUTP (conjugated to fluorescein) nick end labeling (TUNEL) [40]. SKOV-3 cells were treated with *HNTMB *and DNA fragmentation was visualized by fluorescence microscopy (Fig. 4B). To identify the entire population of SKOV-3 cells, the nuclei of cells were counterstained with propidium iodide (PI), a red fluorescence DNA intercalating dye. Treatment of SKOV-3 cells with 0.2 μM *HNTMB *for 24 h revealed an apoptotic population of ~40% of cells with TUNEL positive nuclei (red/green channel overlay resulting in yellow color) whereas the untreated control cells were TUNEL-negative (Fig 4B).

### Induction of apoptosis in ovarian cancer cells after HTMB treatment

**Figure 5:**
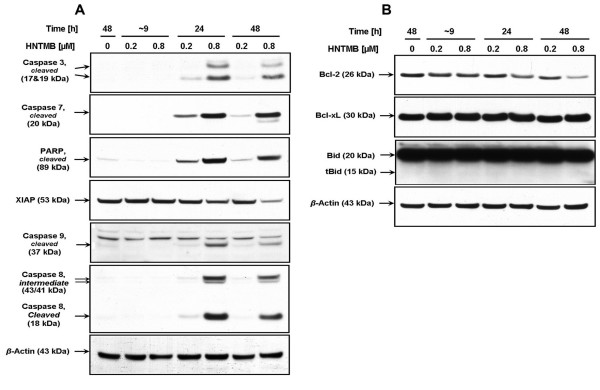
**Expression of apoptotic markers in SKOV-3 cells after *HNTMB *chelator treatment**. SKOV-3 cells were treated with *HMTNB *(0, 0.2, 0.8 μM) for 9, 24 or 48 h. PAGE and Western blot analysis of cell lysates were carried out as described (Materials and Methods). A representative experiment is shown. Immunoblotting was carried out with primary antibodies against PARP, caspase-3, caspase-7, caspase-8, caspase-9, Bcl-2, Bid, and XIAP. As an internal standard for equal loading blots were probed with an anti-β-Actin antibody.

To define the cellular response of SKOV-3 cells upon *HNTMB *treatment we analyzed the activation of apoptotic markers by immunoblotting. Following *HNTMB *treatment for 24 h Western blotting confirmed the activation of effector caspase-3, -7 and inactivation of PARP-1 (involved in DNA repair) in SKOV-3 cells (Fig. 5A). The cleavage of PARP is thought to represent the irreversible stage of cellular process in apoptosis events. In addition the expression of pro-survival marker XIAP, a direct inhibitor of executioner caspases such as caspase-3, was gradually down-regulated within 9 to 48 h following the *HNTMB *treatment (Fig. 5A).

Moreover, it was apparent that *HNTMB *induced both major signaling pathways (intrinsic, extrinsic) described for programmed cell death. A prominent marker for the extrinsic pathway is activation of caspase-8 which occurred following SKOV-3 treatment with *HNTMB *for 24 h (Fig. 5A). Features of the intrinsic pathway include the activation of pro-apoptotic members of the Bcl-2 family and/or down-regulation of anti-apoptotic Bcl-2 family members and cleavage/activation of caspase-9 which also occurred in SKOV-3 following 24 h of *HNTMB *treatment (Fig. 5A). Caspase-9 was activated by a feedback mechanism through cleavage (at Asp330) via caspase-3 yielding a large fragment, p37, (Fig. 5A) and through apoptosome-dependent self-cleavage (at Asp315) yielding fragment p35 (data not shown). Interestingly, down-regulation of anti-apoptotic Bcl-2 but not Bcl-xL was observed (Fig. 5B). In addition, no change in the expression of full-length Bid (22 kD) or truncated-Bid (also known to trigger mitochondrial signaling) was observed (Fig. 5B).

### HNTMB treatment induces SKOV-3 cell cycle arrest in G2/M phase

As described in the previous sections, *HNTMB *is a cytotoxic agent which activates apoptotic processes in SKOV-3 ovarian cancer cells. To investigate if *HNTMB *affects the proliferation of SKOV-3 cells (particularly at drug concentrations when viability is only partially reduced) we analyzed the cell cycle distribution of SKOV-3 cells in response to *HNTMB *treatment by FACS.

**Figure 6:**
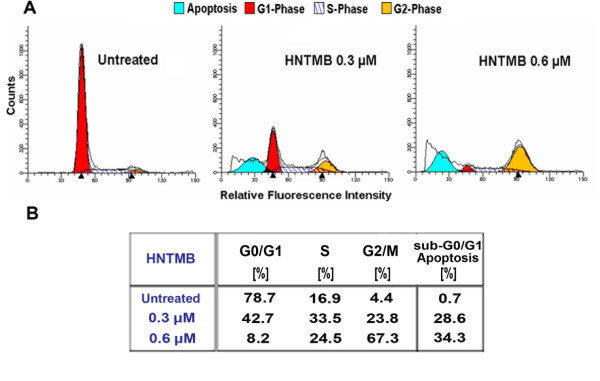
***HNTMB *causes G2-Phase cell cycle arrest in SKOV-3 cells**. SKOV-3 cells were treated with 0.3 or 0.6 μM *HNTMB *for 48 h. Cell cycle analysis of treated and untreated cells was carried out as described (Materials and Methods). Data were obtained by FACS analysis based on propidium-iodide intercalation into cellular chromatin and are presented as **(A) **relative fluorescence intensity in a 2-dimensional FACS profile (ModFit LT software; black lines = data line and model fit line of entire population; shaded areas = model components/subpopulations of G0/G1, S, G2/M, apoptotic cells or as **(B) **a table. Standardized gating was used for all samples. Ten thousand events were analyzed for each sample.

While only 4.4% of untreated cells were present in the G2/M sub-population, *HNTMB *treatment for 48 h led to a dose-dependent G2/M phase arrest (Fig. 6A) with 23.8% cells (at 0.3 μM drug concentration) and 67.3% cells (at 0.6 μM) in G2/M (Fig. 6B). Accordingly, the cell sub-population in G0/G1 phase, when compared to untreated cells (78.7%), was reduced to 42.7% (0.3 μM *HNTMB*) and 8.2% (0.6 μM *HNTMB*), respectively. In addition, FACS analysis after *HNTMB *treatment revealed an increase in the count of apoptotic sub-diploidal/2n cells (sub-G0/G1, Fig. 6B) in a dose-dependent manner. Treatment of this unsynchronized cell culture led to a delayed progression of cells through S-phase (16.9% in untreated SKOV-3) which was apparent at 0.3 μM *HNTMB *(33.5% in S-phase). At the highly cytotoxic concentration of 0.6 μM drug we observed a loss of cells in culture due to cell shrinkage and disintegration, an increase of apoptotic sub-diploidal/2n cells (34.3%) while 24.5% of the cells were in S-phase.

### HNTMB/Copper complexes but not HNTMB/Iron complexes mediate cytotoxicity and ROS generation

*HNTMB *as a chelator has the potential to bind various trace metals present in human tissues, cancer, serum, plasma and cell lines, such as iron, copper, zinc, magnesium, cadmium. In a viability assay we determined the cytotoxicity of *HNTMB *when complexed with Fe(II), Fe(III), Cu(I) or Cu(II). Complexes were formed by combining stock solutions of *HNTMB *and respective metal salts (FeCl_2_, FeCl_3_, CuCl or CuCl_2_) to 1000× the desired final assay concentration in DMSO, followed by incubation at 37°C for 30 min before further dilution in complete medium for cell treatment.

**Figure 7:**
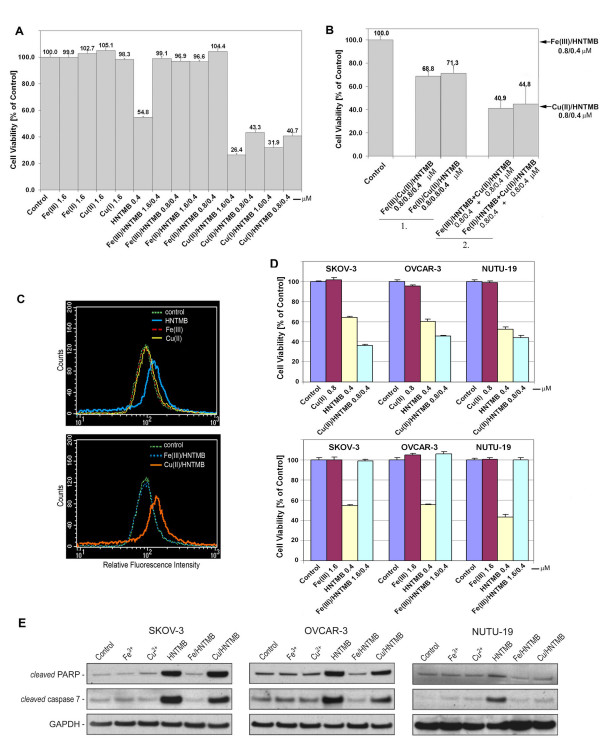
**Effect of iron/*HNTMB *and copper/*HNTMB *complexes on viability, ROS generation, and induction of apoptosis**. (A) Cytotoxicity of *HNTMB *in SKOV-3 cells when complexed with Fe(II), Fe(III), Cu(I) or Cu(II) Complexes were formed by combining *HNTMB *and respective metal salts to 1000× [0.4 mM *HNTMB*; 1.6 or 0.8 mM Fe(II), Fe(III), Cu(I) or Cu(II)] the assay concentration before addition (diluted 1:1000) to the cells (48 h treatment). As controls served cells left untreated, incubated with metal salts (1.6 μM) or non-complexed *HNTMB *(at IC_50 _concentration, 0.4 μM). Data are expressed as mean of triplicate determinations (X ± SD) in % cell viability of untreated cells [100%]. (B) Combinational effect of *HNTMB *iron- and copper-complexes on cell viability in SKOV-3 cells. *Group 1: HNTMB *complex formation was performed under competing conditions for Fe and Cu-chelation at 1000× the desired assay concentration before addition of this mixture (diluted 1:1000) to the cells (48 h treatment). ***Group 2: ***Stock solutions (1000×) of pre-formed complexes of Fe(II) or Fe(III)/- and Cu(II)/*HNTNB *were individually diluted before simultaneous addition to the cells (48 h treatment). (C) Detection of intracellular ROS in SKOV-3 cells Generation of ROS following treatment with non-complexed or complexed *HNTMB *was measured by flow cytometry. Data are presented as relative-fluorescence-intensity in a 2-dimensional FACS profile (standardized gating; 10.000 events). (D) Effect of *HNTMB *complexation with Fe(III) or Cu(II) on the viability of various ovarian cancer cell lines SKOV-3, OVCAR-3 or NUTU-19 cells were treated with 0.4 μM non-complexed or complexed (Cu(II)-upper panel; Fe(III)-lower panel) *HNTMB *for 48 h and the assay carried out. (E) Effect of *HNTMB *complexation with Fe(III) or Cu(II) on expression of apoptotic markers in various ovarian cancer cell lines SKOV-3, OVCAR-3 or NUTU-19 cells were treated with 0.4 μM non-complexed or complexed *HNTMB *for 48 h. Western blot analysis of cell lysates was carried with primary antibodies against PARP-1, caspase-7 and GAPDH (control).

The IC_50 _following 48 h treatment of SKOV-3 with non-complexed *HNTMB *is approximately 0.4 μM. Fig. 7A shows that at this drug concentration cell viability was reduced to 54.8% of the untreated controls. Previously, in a publication on the chemotherapeutic activity of Salophene complexes we showed that Fe[III] alone does not display significant cytotoxic effects in SKOV-3 cells at or below the concentrations of 60 μM, whereas Cu[II] displays partial cytotoxicity (33% of cells) at a concentration of 60 μM but not ≤30 μM [41]. Accordingly, treatment of SKOV-3 cells with FeCl_2_, FeCl_3_, CuCl or CuCl_2 _at concentrations of 1.6 μM did not affect cell viability nor did 0.4 μM *HNTMB *when complexed with Fe(II) or Fe(III). In contrast, 0.4 μM *HNTMB *when complexed with Cu(I) or Cu(II) was highly cytotoxic to the cells (Fig. 7A). When *HNTMB *was chelated with Cu(II) (at 1000× the desired assay concentration and prior to 1000 fold dilution in complete medium for treatment), cell viability was reduced 2-fold (26.4%; Cu(II)/*HNTMB *1.6/0.4 μM) as compared to non-complexed *HNTMB *(54.8%). Using a Cu(I)/*HNTMB *complex (1.6/0.4 μM) viability was reduced to 31.9%. When chelation was carried out in the presence of Cu salts cytotoxicity was still higher (43.3% for Cu(II)/*HNTMB *0.8/0.4 μM and 40.7% for Cu(I)/*HNTMB *1.6/0.4 μM) compared to non-complexed *HNTMB*. These results indicated that (i) we efficiently achieved *HNTMB*-metal complexation under the experimental conditions described (Material and Methods), (ii) *HNTMB *pre-chelated with iron did not exert cytotoxic effects in SKOV-3 cells, (iii) no significant dissociation of these pre-formed complexes occurred under our experimental conditions because measured cytotoxicities were, for example, less than [in the case of Fe (II or III)] that for non-complexed *HNTMB*, and iv). Pre-Cu chelated *HNTMB *was more cytotoxic than non-complexed *HNTMB*.

Next, we carried out *HNTMB *complex formation under competing conditions for Fe and Cu-salts (Fig. 7B; group 1) prior to performing cell viability assays or we combined pre-formed Fe/- and Cu/*HNTMB *complexes and measured their effects in the viability assay (Fig. 7B; group 2.). When Fe(II)- and Cu(II)-salts were incubated together with *HNTMB *prior to treatment (Fig. 7B, group 1), cell viability was reduced to 71.3% (Fe(II)/Cu(II)/*HNTMB*) compared to 104.4% for Fe(II)/*HNTMB*, 43.3% for Cu(II)/*HNTMB *and 54.8% for non-complexed *HNTMB *(Fig. 7A). These data suggest that *HNTMB*, under our experimental conditions, had a similar affinity for Fe salts and Cu salts. Accordingly, when Fe(III) and Cu(II) were incubated together with *HNTMB *prior to treatment viability was reduced to 68.8% for Fe(III)/Cu(II)/*HNTMB*) compared to 96.9% for Fe(III)/*HNTMB*, 43.3% for Cu(II)/*HNTMB *and 54.8% for non-complexed HNTMB (Fig. 7A).

When Fe or Cu complexes were pre-formed (0.8 μM metal, 0.4 μM *HNTMB*) and added individually to the cells (Fig. 7B, group 2), cell viability was 40.9% for Fe(III)/*HNTMB*+Cu(II)/*HNTMB *and 44.8% for Fe(II)/*HNTMB*+Cu(II)/*HNTMB*) instead of 43.3% viability when the same concentration of Cu(II)/*HNTMB *alone was used (Fig. 7B, arrow). This suggested that the cytotoxicity exerted by a copper/*HNTMB *complex was not altered and that no additional cytotoxic effect through co-treatment with an iron/*HNTMB *complex was achieved.

One potential strategy suggested to treat cancer is to generate an excess amount of ROS in tumor tissue to induce necrosis and/or apoptosis. We determined if SKOV-3 following treatment with non-complexed *HNTMB *or Cu/Fe complexed-*HNTMB *lead to the generation of ROS. These species were detected via Carboxy-H2DCFDA, which is a fluorescein derivative that is cell-permeable and non-fluorescent. In the presence of a cellular oxidant, the molecule is oxidized and produces green-fluorescence that is detected by flow cytometry. As shown in Fig. 7C, ROS generation in SKOV-3 cells remained unchanged as compared to untreated controls when either Fe(III)/*HNTMB *complex (0.8/0.4 μM) or FeCl_3 _and CuCl_2 _metal salts (0.8 μM) were used. However, ROS generation increased (shift in relative fluorescence intensity, Fig. 7C) following treatment of cells for 20 h with either 0.4 μM non-complexed *HNTMB *(upper panel) or Cu(II)/*HNTMB *(0.8/0.4 μM; lower panel) which correlated with a reduction in cell viability by these compounds at the same concentration (Fig. 7A).

To rule out that the effects of *HNTMB *complexation are cell type specific we compared the cytotoxic effects (Fig. 7D) and induction of apoptotic markers (Fig. 7E) by *HNTMB *when complexed with Fe(III) or Cu(II) in three different ovarian cancer cell lines.

Human platinum resistant OVCAR-3 cells as well as rat NUTU-19 displayed a similar response profile as SKOV-3 and treatment of these cell lines with CuCl_2 _at concentrations of 0.8 μM did not affect cell viability. In contrast, 0.4 μM *HNTMB *when complexed with Cu(II) was highly cytotoxic to the cells and exceeded toxicity of non-complexed *HNTMB *(Fig. 7D, top panel). Remarkably, complexation of *HNTMB *with Fe(III) fully prevented cytotoxicity of the compound. The viability of SKOV-3, OVCAR-3 and NUTU-19 compared to that of untreated controls or cells treated with the respective Fe(III) salt (Fig. 7D, bottom panel).

Following *HNTMB *treatment Western blotting confirmed the activation of effector caspase-7 and inactivation of PARP-1 (involved in DNA repair) in all three cell lines by 0.4 μM of either non-complexed *HNTMB *or when complexed with Cu(II) (Fig. 7E). Apoptosis was not induced through *HNTMB *when complexed with Fe(III) and expression levels of activated caspase-7 and inactivated PARP-1 were similar to the controls (untreated cells or cells treated with FeCl_3 _or CuCl_2_) (Fig. 7E). Apoptosis was induced more rapidly by the copper-complex as compared to treatment with non-complexed *HNTMB *with a higher number of cells showing degradation (data not shown). In line with the kinetic study on the expression of apoptotic markers peaking at 24 h (Fig. 5) activation of caspase-7 or inactivation of PARP-1 at 48 h treatment with Cu(II)/*HNTMB *when compared to treatment with non-complexed *HNTMB *was past the peak and less pronounced. However, the 48 h incubation was chosen to perform the Western Blotting analysis (Fig. 7E) in conjunction with our standardized Viability assay (48 h incubation; Fig. 7A, B, D) and ROS detection assay (Fig. 7C) all reveal that complexation of *HNTMB *with copper but not with iron results in a potent drug to treat ovarian cancer cells.

## Discussion

### Chelators in cancer treatment

A low overall survival for advanced stages of ovarian cancer is related to the appearance of drug resistance to standard agents, notably organometallic platinum compounds. Research has been directed at the discovery of non-platinum chemotherapeutics with increased activity and response rates and among them chelating drugs and chelator metal complexes [7,41-44]. In addition, the efficacy of current anti-cancer drugs may be increased by combinational treatment with chelating drugs which have been shown to affect the metabolism and toxicity of anti-cancer compounds such as adriamycin, mitozantrone, bleomycin and hydroxyurea (HU) [7].

The objective of the present study was to investigate the potential of six different lipophilic aroylhydrazone chelators (*AHC*) including novel compound *HNTMB *as anti-tumor drugs and to analyze modes of action leading to cell death of ovarian cancer cells following *HNTNB *treatment. As a model system we choose OVCAR-3 cells resistant to clinically relevant concentrations of adriamycin, melphalan and cisplatin and SKOV-3 cells resistant to several cytotoxic drugs including cisplatin and adriamycin (see ATCC, Manassas, VA; http://www.atcc.org). To set the stage for potential future *in vivo *experiments using chelators in a syngenic rat tumor model [41] we included rat ovarian NUTU-19 cancer cell line in a screen for cytotoxicity by the various *AHC *tested here.

### Efficacy of aroylhydrazone chelators in cancer cells

Previous work showed that AHCs (often referred to as iron-chelators due to initial studies on high Fe chelation efficacy in cell lines and *in vivo *models) [45] exert anti-proliferative and/or cytotoxic effects in neoplastic cells including cell lines derived from bladder carcinoma, rat hepatoma, T-cell leukemia, neuroblastoma, melanoma and leukemia [34,35,45-47]. To date no studies on the effect of *AHC *compounds in ovarian cancer models, neither *in vitro *nor *in vivo*, have been published, and their mechanism of action leading to cell death remains unknown.

The present study shows that novel compound *HNTMB *in its cytotoxic capacity (IC_50 _between 200-400 nM depending on the ovarian cancer cell line) is superior to other *AHC *compounds (oVtBBH, HNtBBH, StBBH/206, HNTh2H/315 and HNI/311 and *HNTMB*) (IC_50 _0.8-6 μM) as well as to deferoxamine (DFO) (IC_50 _12-25 μM) which was included as a reference. StBBH/206, HNTh2H/315 and HNI/311 in previous studies displayed anti-proliferative effects in neuroblastoma cell lines (IC_50 _= 0.2-1.2 μM), melanoma cells (IC_50 _0.8-1.0 μM) and leukemia cells (IC_50 _0.4-1.3 μM) [34,35]. Two of the three AHC (HNTh2H/315 and HNI/311) and *HNTMB *possess a 2-hydroxy-1-naphthylaldehyde moiety. The high lipophilicity of this aldehydic moiety when compared to *AHC *compounds with a less lipophilic pyridoxal or salicylaldehyde group is correlated to increased anti-proliferative effects [34] and likely contributes to the effectiveness of the novel *HNTMB *studied here. *HNTMB*, apart from a hydroxy-1-naphthylaldehyde moiety and unlike the other structurally-related *AHCs *studied here (HNI/311, HNTh2H/315, HNtBBH), possesses a trimethoxybenzene moiety, which is found in a large number of biologically active compounds, including anti-cancer drugs such as combretastatin. The trimethoxybenzene moiety in *HNTMB *may be associated with the further improvement of its cytotoxicity in comparison to the structurally-related chelators without such a functional group. However, further studies must be conducted to prove whether this moiety is directly responsible for the high degree of cytotoxicity produced by *HNTMB*. The control compound DFO, structurally unrelated to *AHCs*, is a naturally occurring hexadentate iron-chelator that has been used as a therapeutic agent against ovarian [20] and other cancer cells [48-53]. However, the hydrophilic property of DFO limits its membrane-permeability and efficacy to target the intracellular trace metal pool including iron [54] or affect other intracellular processes. In contrast to DFO, compounds such as *HNTMB *display a greater potency because of their better membrane-permeability profiles.

The observation to cytotoxic effects of *HNTMB *on SKOV-3, OVCAR-3 and NUTU-19 ovarian cancer cells led to a screen performed by the NCI-DTP against a panel of cell lines http://dtp.nci.nih.gov/screening.html derived from human tumors of different origin. It became apparent that *HNTMB *displayed potent cell line-specific but not tumor type-specific cytotoxicity with GI_50 _values in a 240-fold range (between 10 nM and 2.4 μM depending on the cell line studied). Thus, cell death, depending on the target tumor or cancer cell line, can be induced by *HNTMB *at concentrations in the nanomolar range. At these concentrations *AHCs *may not exert their effects by critical depletion of the pool of intracellular iron but by binding to other trace metals such as copper (see discussion below). *In vivo *experiments have shown that effective treatment of tumors with related thiosemicarbazone Dp44mT, which reduced growth in melanoma xenografts in nude mice by 92%, required low doses that did not cause iron depletion [55]. It is noteworthy that no direct correlation between the anti-proliferative activity of *AHCs *with their abilities to prevent iron-uptake or mobilization in neuroblastoma cells was reported [34] suggesting that interference with iron metabolism may not be responsible for the effect of *AHCs *on cell viability. Early studies examined the use of copper complexes of aroylhydrazone derivatives as therapeutic agents; Cu(II) complexation of SBH not only increased the compounds cytotoxicity but administration of this complex in mice was well tolerated [46]. The present study revealed that *HNTMB *at 400 nM when complexed with Cu(I) or Cu(II) displayed a high cytotoxicity correlated with massive generation of ROS. In contrast, *HNTMB *complexed with Fe(II) or Fe(III) lost its cytotoxicity and did not alter basal levels of ROS in SKOV-3 cells. Non-complexed *HNTMB *displayed partial cytotoxicity and by binding to various trace metals is likely to disturb a multitude of functions important for cell proliferation and survival. HNTMB, as a tridentate coordinating ligand, has the ability to bind copper or iron in a stochiometric ratio of 1:1. Its IC50 determined for ovarian cancer cell lines is 200-400 nM (75-150 μg/L) and the assay concentration of non-complexed HNTMB that we used was 150 μg/L which is a fraction of the total iron or copper content of cells. Ovarian cancer tissues contain 0.3-0.7 mg/kg copper and 15-17 mg/kg iron content [10]. By binding intracellular copper HNTMB can create a toxic ROS-generating complex, while intracellular iron-chelation by *HNTMB *depending on its concentration may lead to a variety of cellular consequences previously described for aroylhydrazone chelators [31]. These include ribonucleotide-reductase/RR inhibition, redox-activity leading to the hydroxylation of benzoate and the degradation of DNA in the presence of Fe(II) and H_2_O_2_, down-regulation of cell cycle regulators, and activation of WAF (mediator of p53 tumor suppression) among other genes.

### Response mechanism of cancer cells to HNTMB treatment

Through various experimental approaches the present study suggests that the mode of action of *HNTMB *in SKOV-3 cells relies on a variety of inter-dependent processes such as generation of ROS, DNA degradation, induction of apoptosis, and arrest of cell cycle progression through G2/M phase. ROS are generated in SKOV-3 when treated with a copper/*HNTMB *complex but not when an iron/*HNTMB *complex was used. Thus, ROS generation observed after treatment with non-complexed *HNTMB *is, at least, partially due to the drug binding intracellular copper (possibly by binding to other trace metals with a similar result). Production of ROS results in apoptosis and/or necrosis and can be used for selective targeting of tumor cells which possess higher oxidative stress level and display alteration of antioxidant enzymes (catalase, SOD) as compared to normal cells. Apparently, the Cu/*HNTMB *complex, as suggested for other chemotherapeutic drugs, may be used in oxidation therapy by elevating H_2_O_2 _and superoxide radical in tumor cells above the survival/death threshold (see introduction) [17,56]. Even though excess copper is known to be a potent oxidant causing the generation of ROS, we ruled out that unbound copper present in the sample of the Cu/*HNTMB *complex could be responsible for ROS generation. In controls, neither Cu(I) nor Cu(II) alone, at concentrations of 1.6 μM, resulted in cytotoxicity or ROS production during treatment. Previously, we determined that treatment with non-chelated copper does not affect the viability of ovarian cancer cell at or below a concentration of 30 μM [41]. Therefore, we suggest that future studies should focus on the mechanistic responses of tumor cells to treatment with copper/*AHCs *complexes in general and specifically with a Cu/*HNTMB *complex in ovarian cancer models. The mechanism(s) by which the copper-HNTMB complex generates elevated ROS levels remains under investigation but may include targeting of cellular thiol-containing anti-oxidant molecules such as glutathione (GSH) as this has been shown for other copper complexes such as CuNG [57]. Even upon treatment with non-complexed *HNTMB *intracellular copper-chelation by *HNTMB *might selectively be used to cause ROS-mediated cell death in certain tumors because a higher copper level is apparent both in serum and tumors of cancer patients including those suffering from breast and ovarian cancer [8-10]. Reference values determined by Yaman *et al*. [10] showed an average copper content in malignant ovarian tissue of 0.7 mg/kg copper versus 0.3 mg/kg in benign ovarian tissue. Interestingly, in these tissues the iron content for cancerous and non-cancerous conditions is comparable (15-17 mg/kg) [10]. Thus, the iron/copper ratio decreases by a factor of 2 from benign to malignant ovarian tissues and may represent a target for chelation therapy.

In the present study, apart from ROS generation by ovarian cancer cells upon treatment with *HNTMB*, we observed other cellular responses of SKOV-3 cells to this chelator such as DNA degradation, arrest of cell cycle progression in G2/M phase and activation of apoptotic signaling. Cell cycle regulation is known to be a target of chelating agents and has been attributed to the depletion of intracellular iron. Chelators of the *AHC *class can regulate the expression of various proteins involved in cell-cycle control such as CDK2, cyclins A, B1, D1, D2, D3, *WAF1 *(inhibitor of CDKs) as shown for HNI/311 in neuroblastoma and an erythroleukemia cell line. While chelators such as HNI/311 or DFO generally block G1/S phase transition, effects on the G2/M transition during the cell cycle has also been implicated [58,59]. For DFO and a 3-hydroxypyridin-4-one iron-chelator, it has been shown in K562 erythroleukemia cells that at higher concentrations these cells undergo arrest in G1/S while at lower drug concentrations they accumulated in G2 and M phase without an effect on DNA synthesis [60]. Thus, cell cycle regulation through iron-chelators may depend on the dose and the cell line studied. *HNTMB *at 600 nM lead to an accumulation of SKOV-3 primarily in G2/M phase. Future studies, beyond the scope of the present work, could focus on cell cycle checkpoints affected by *HNTMB *treatment in synchronized cancer cells and verify the general concept that iron-chelation (and not depletion of other intracellular metals) is responsible for these effects. Generally, targeting cell cycle key regulators has been suggested as a supplemental approach to anti-cancer therapies [61-64]. In addition, it is noteworthy that cells are most radiosensitive in the G2/M phase [65]. Based on this and our findings, *HNTMB *could be used as a radiosensitizer.

Drug treatment leading to programmed cell death (Apoptosis) results in the activation of *initiator *caspases which upon activation subsequently activate downstream *effector *caspases that are responsible for the cleavage of many intracellular proteins, leading to the morphological and biochemical changes associated with apoptosis [66,67]. *HNTMB *treatment of SKOV-3 cells resulted in strong activation/cleavage of *initiator *caspase-8 and -9 and of *effector *caspase-3 and-7, while PARP-1 (involved in DNA repair) [68] was inactivated/cleaved following drug treatment. It is noteworthy that *HNTMB *down-regulated expression of the X-linked inhibitor of apoptosis (XIAP). Down-regulation of XIAP expression in ovarian cancer cells results in apoptosis *in vitro *and prolonged survival of ovarian cancer-bearing mice, which indicate that XIAP may be a valuable therapeutic target in ovarian cancers [69]. Interestingly, cisplatin-resistance in human ovarian surface epithelial (hOSE) cancer cells is correlated with the inability of cisplatin to down-regulate XIAP expression [70]. *HNTMB *may be an alternative apoptosis inducing drug in certain platinum-resistant cancers via XIAP reduction as shown to occur in SKOV-3 ovarian cancer cells used in the present study.

*HNTMB *induced both major signaling pathways (*intrinsic, extrinsic*) as evidenced by the activation of initiator caspases similar to the effect of other iron chelators such as tachpyridine, DFO, and dipyridyl [71]. *Intrinsic *pathway activation may have mitochondrial damage as a pre-requisite, leading to the activation of pro-apoptotic members of the Bcl-2 family and/or inactivation of anti-apoptotic Bcl-2 and results in the mitochondrial release of cytochrome C which in turn activates *initiator *caspase-9 [72] as seen in SKOV-3 cells following *HNTMB *treatment. It has previously been suggested that the mitochondrial pathway takes a center-role in iron chelator-mediated cell death since over-expression of anti-apoptotic Bcl-2 and Bcl-XL promotes cell (in HeLa cervix carcinoma cells) survival and chelator-mediated cell death can be blocked by a dominant-negative caspase 9 and Bcl-XL over-expression [71]. We show here that the exclusive down-regulation of Bcl-2 expression (in contrast to unmodified Bcl-XL or Bid) takes a center role in the pro-apoptotic response of SKOV-3 cells to treatment with *HNTMB*. Similarly, other *AHC *compounds and their iron complexes cause apoptosis via the mitochondrial pathway (in Jurkat T cells and K562 leukemia) which could be counteracted by Bcl-2 overexpression [73]. Because anti-apoptotic Bcl-2 is highly expressed in various human cancers, this feature of *HNTMB *increases its potential as an alternative anti-tumor drug. Bcl-2 also mediates the resistance of cancers to conventional therapies such as radio- and chemo-therapy. Thus, the blockage of Bcl-2 protein expression by *HNTMB *treatment could be useful to sensitize cancer cells to conventional therapies [74]. A COMPARE analysis using the NCI's anticancer drug screen database suggested the involvement of Bcl-2 protein as a putative target of *HNTMB*. in its mechanism of action. The COMPARE computer program utilizes cytotoxicity data derived from screening compounds against 60 human cancer cell lines to calculate the Pearson correlation coefficient (positive correlation of 0.45 for *HNTMB*), between the data for the seed compound and those for past agents in the database to identify similar molecular targets or similar mechanisms of resistance [75-79].

Induction of apoptosis by chelating agents, including representatives of *AHCs*, has been primarily associated to their capability to bind/deplete intracellular iron [73]. These properties may be especially relevant for cancer treatment because (i) the intracellular concentration of iron is generally higher than that of copper and (ii) it can be significantly elevated in both serum and tumor tissue of cancer patients with endometrial or breast cancer [9,10]. The pro-apoptotic effects of non-complexed *HNTMB *at higher concentrations may be partially linked to its capacity to bind/deplete intracellular iron. However, it is apparent that a copper/*HNTMB *complex at a concentration of 0.4 μl strongly induces apoptosis, while an iron/*HNTMB *complex has no such effects. Observations related to the role of organocopper complexes in apoptotic events include disruption of the peroxide and thiol metabolism with subsequent up-regulation of pro-apoptotic Bcl-2 family members (Bak/Bax in melanoma and epithelial carcinoma cells) [80]. Previous studies have revealed that in the presence of complexed copper (Cu-NTA) the expression of anti-apoptotic Bcl-2 and Bcl-XL can be down-regulated (HL-60 cells) [81]. Accordingly, the pro-apoptotic activity of *HNTMB *may include direct effects on Bcl-2 regulation when complexed with copper in contrast to the postulated indirect pro-apoptotic effects linked to intracellular iron depletion. We conclude that non-complexed *HNTMB *exerts cytotoxicity on ovarian cancer cells in a dual function by binding copper [Cu(I) and Cu(II)] present in the human body [82] and the intracellular iron present in the Fe(II) and Fe(III) states [31].

## Conclusions

The present report suggests that *HNTMB *displays properties akin to an anti-cancer drug and could be an alternative to platinum derivatives in the treatment of ovarian cancer and other solid tumors. *HNTMB *can chelate iron and copper of different oxidation states and possess anti-cancer drug attributes via its properties as a chelator of intracellular trace-metals or, alternatively, as a cytotoxic organic metal- (e.g., copper) complex. *HNTMB *and other chelating drugs of the lipophilic aroylhydrazone class may prove to be superior to other chelators or metal complexes already in use for the treatment of cancer. Experimental approaches using the ovarian cancer cell line SKOV-3 as a model system suggest various modes of action exerted by *HNTMB *based on the generation of ROS, caspase activation, Bcl-2 down-regulation, DNA degradation and G2/M phase cell cycle arrest.

## List of abbreviations used

AHC: aroylhydrazone chelators; DAPI: 4',6-diamidino-2-phenylindole; DFO: deferoxamine; DMEM: Dulbecco's Modified Eagle's Medium; DMSO: dimethyl sulfoxide; FACS: fluorescence activated cell sorter; FL: fluorescein; H2DCFDA: 2',7'-dichlorodihydrofluorescein diacetate; MTS: 3-(4,5-dimethylthiazol-2-yl)-5-(3-carboxymethoxyphenyl)-2-(4-sulfophenyl)-2H-tetrazolium; NMR: nuclear magnetic resonance; PBS: phosphate buffered saline; ROS: reactive oxygen species; RR: ribonucleotide reductase; SOD: superoxide dismutase; TCA: trichloroacetic acid; TUNEL: terminal deoxynucleotidyl transferase dUTP nick end labeling.

## Competing interests

The authors declare that they have no competing interests.

## Authors' contributions

KK and RKS performed the experimental procedures with support from TSL. KK, TSL and LB were responsible for experimental design, interpretation of the results and writing the manuscript. All authors read and approved the final manuscript.

## Pre-publication history

The pre-publication history for this paper can be accessed here:

http://www.biomedcentral.com/1471-2407/10/72/prepub
